# Supplementary material for: HIV-seq reveals gene expression differences between HIV-transcribing cells from viremic and suppressed people with HIV
Source: Nat Commun. 2026 Mar 3;17:1540. doi: 10.1038/s41467-026-68797-3 (PMC12957429; doi:10.1038/s41467-026-68797-3)
Supplement: Supplementary file 1 — Supplementary Information [file 41467_2026_68797_MOESM1_ESM.pdf]

SUPPLEMENTARY FIGURES

Figure S1

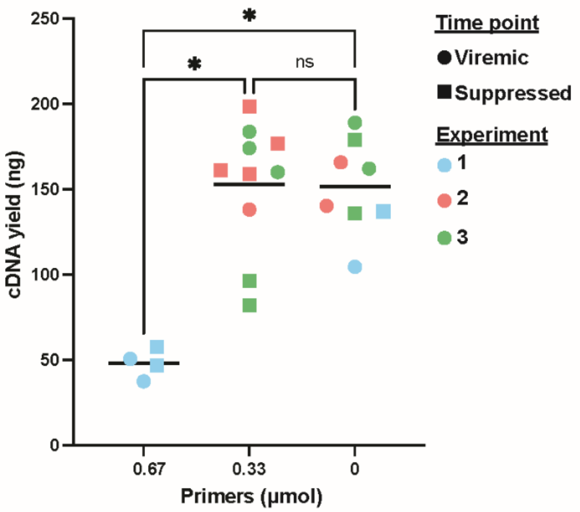

Figure S1. The effects of HIV primer concentration on cDNA recovery. Total cDNA recovery in the absence versus presence of HIV primers, demonstrating the poor cDNA recovery upon addition of 0.67 µM but not 0.33 µM of primers. Data from viremic (circles) and suppressed (squares) samples from three PWH (1, 2, and 3) are shown. Horizontal lines represent median values. P=0.0107 for 0.67 µM vs. 0.33 µM and P=0.0206 for 0.67 µM vs. no primers, as determined using a Kruskal-Wallis test (two-tailed test). Source data are provided as a Source Data file.

Figure S2

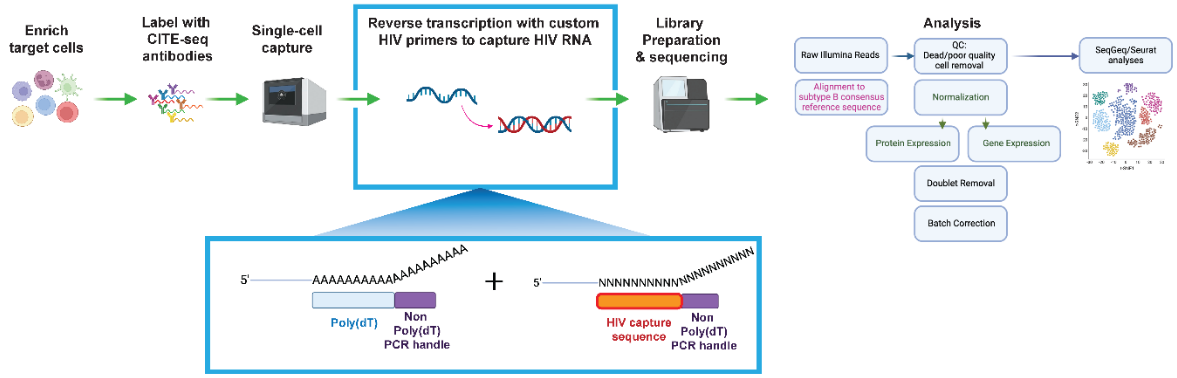

Enrich target cells
Label with CITE-seq antibodies
Single-cell capture
Reverse transcription with custom HIV primers to capture HIV RNA
Library Preparation & sequencing
Analysis
Raw Illumina Reads
QC: Dead/poor quality cell removal
SeqGeq/Seurat analyses
Alignment to subtype B consensus reference sequence
Normalization
Protein Expression
Gene Expression
Doublet Removal
Batch Correction
5'
AAAAAAAAAAAAAAAAAAAA
Poly(dT)
Non Poly(dT) PCR handle
+
5'
NNNNNNNNNNNNNNNNNNNN
HIV capture sequence
Non Poly(dT) PCR handle

Figure S2. HIV-seq method to increase detection of HIV transcripts by single-cell sequencing.

Schematic of HIV-seq protocol. PBMCs from PWH are enriched for CD4+ T cells and then labeled with CITE-seq antibodies to enable subsequent surface phenotyping. During cell encapsulation using a 10X Chromium instrument, custom-designed HIV-specific capture oligos described in Fig. 1A that have been appended to a non-poly(dT) PCR handle are spiked in with the poly(dT) oligos and incorporated into the 10X Genomics' Chromium Next GEM Single Cell 5' workflow. Both gene expression (GEX) and antibody-derived tag (ADT, for CITE-seq) libraries are processed through the standard 10X workflow and then sequenced. Data analysis was performed using the Seurat pipeline, SeqGeq software, and custom scripts. Created in BioRender. Telwatte, S. (2025) https://BioRender.com/0p3e5zo.

Figure S3

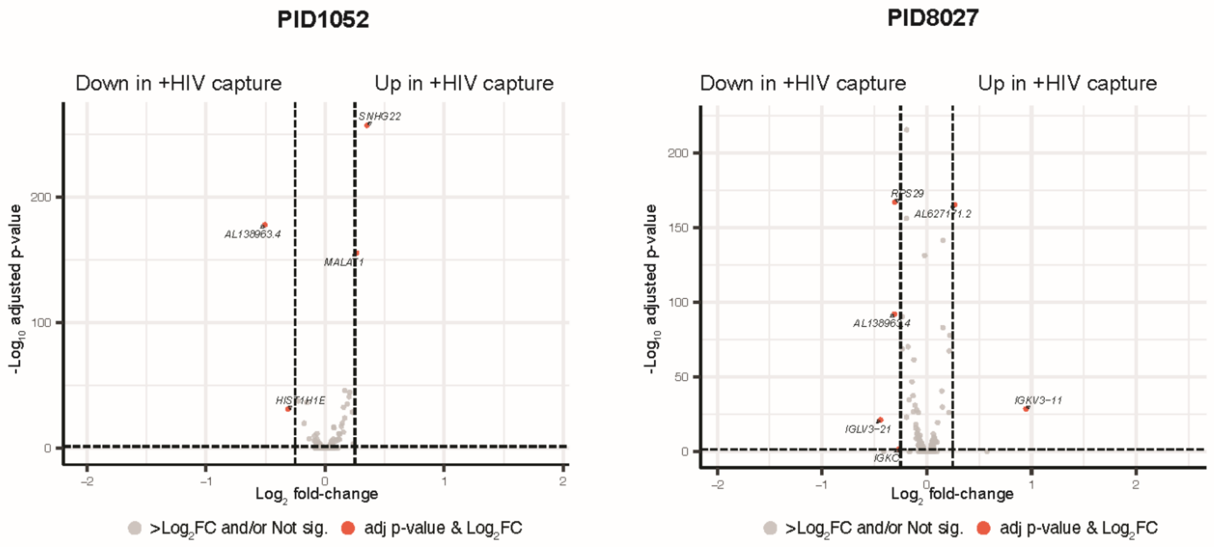

PID1052
PID8027
Down in +HIV capture
Up in +HIV capture
-Log10 adjusted p-value
Log2 fold-change
SNHG22
AL138963.4
MALAT1
RPS29
IGKV3−11
IGLV3−21
IGKC
>Log2FC and/or Not sig.
adj p-value & Log2FC

Figure S3. HIV-seq minimally impacts the profile of the host cellular transcriptome analyzed by scRNA-seq. Volcano plots displaying differentially expressed genes (DEGs) for the two viremic individuals (PID1052 and PID8027) processed in the absence vs. presence of HIV capture sequences. Significantly differentially up- and down-regulated transcripts are annotated. Red dots correspond to genes with $\geq 0.25 \log_2$ fold-change expression and p value <0.05 (adjusted for multiple comparisons), as determined by the two-sided Wilcoxon rank sum test.

Figure S4

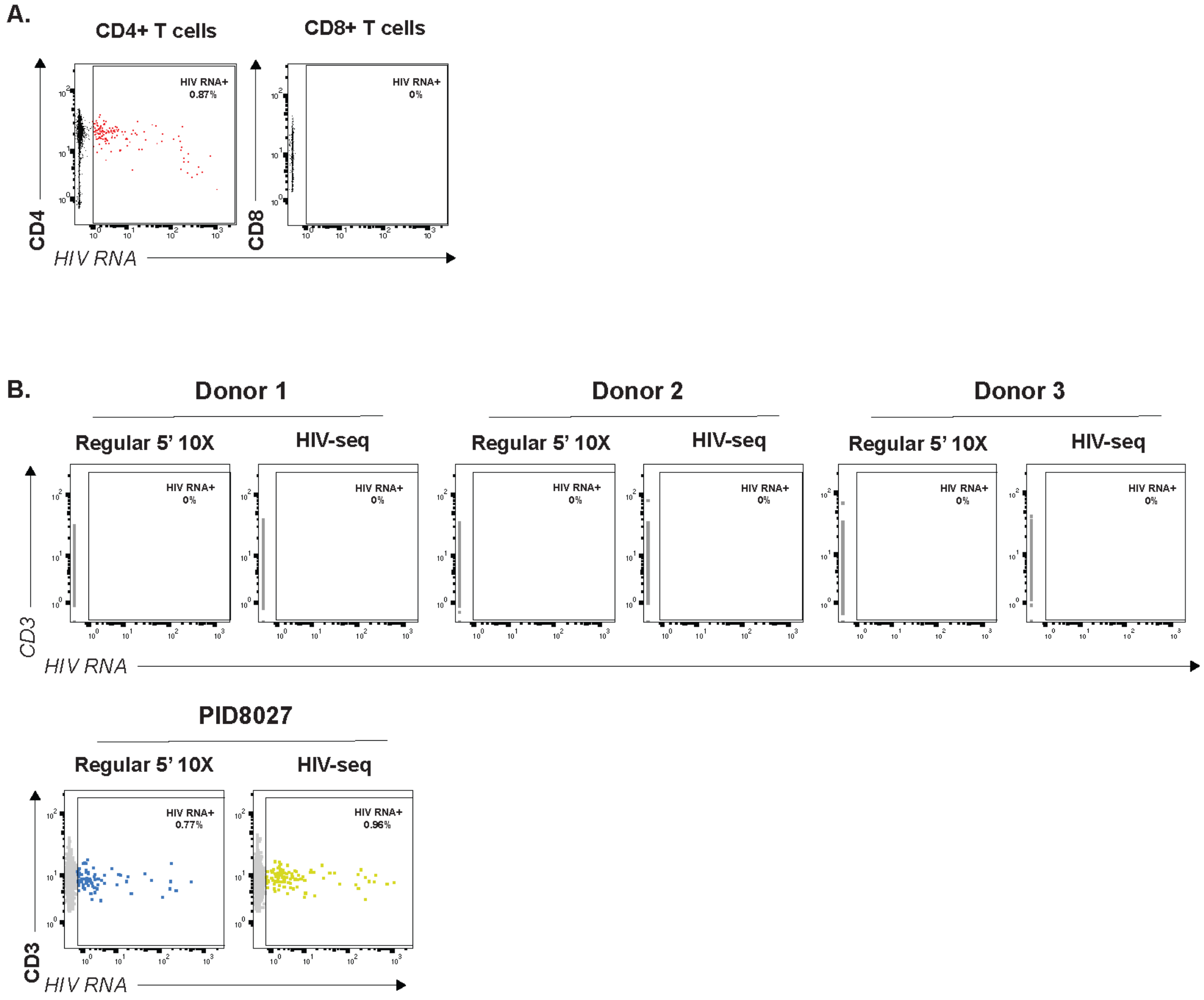
A.
CD4+ T cells
CD8+ T cells
HIV RNA+
0.87%
HIV RNA+
0%
CD4
CD8
HIV RNA
B.
Donor 1
Donor 2
Donor 3
Regular 5' 10X
HIV-seq
HIV RNA+
0%
CD3
HIV RNA
PID8027
Regular 5' 10X
HIV-seq
HIV RNA+
0.77%
HIV RNA+
0.96%
CD3
HIV RNA

Figure S4. HIV-seq does not result in false-positive identification of HIV RNA+ cells from CD8+ T cells during viremia or CD4+ T cells from uninfected donors. A. No HIV RNA+ cells are detected from CD8+ T cells during viremia. CD45+CD3+ T cells analyzed by HIV-seq were gated for CD8- cells (left, to account for potential CD4+ T cells that may have downregulated cell-surface CD4) and CD8+ cells (right), and visualized for CD4 and CD8 expression as indicated along with levels of HIV transcripts. Shown are representative results from one of four viremic PWH. **B.** PBMCs from three HIV-uninfected donors (Donors 1, 2, and 3) were subjected to the regular 5' 10X Genomics scRNA-seq platform or HIV-seq. The total numbers of cells sequenced were as follows: Donor 1: Regular = 4,668 cells, HIV-seq = 7,260 cells; Donor 2: Regular = 5,417 cells, HIV-seq = 5,998 cells; Donor 3: Regular = 12,327 cells, HIV-seq = 8,753 cells. The plots display CD3 expression (y-axis) and HIV transcripts (x-axis). In all three uninfected donors, HIV RNA+ cells were not detected. For comparison, we show the corresponding data from viremic donor PID8027, with cells pooled from two wells (Regular = 12,248 cells, HIV-seq = 14,640 cells), where HIV RNA+ cells were detected both in the absence and presence of HIV capture oligos.

Figure S5

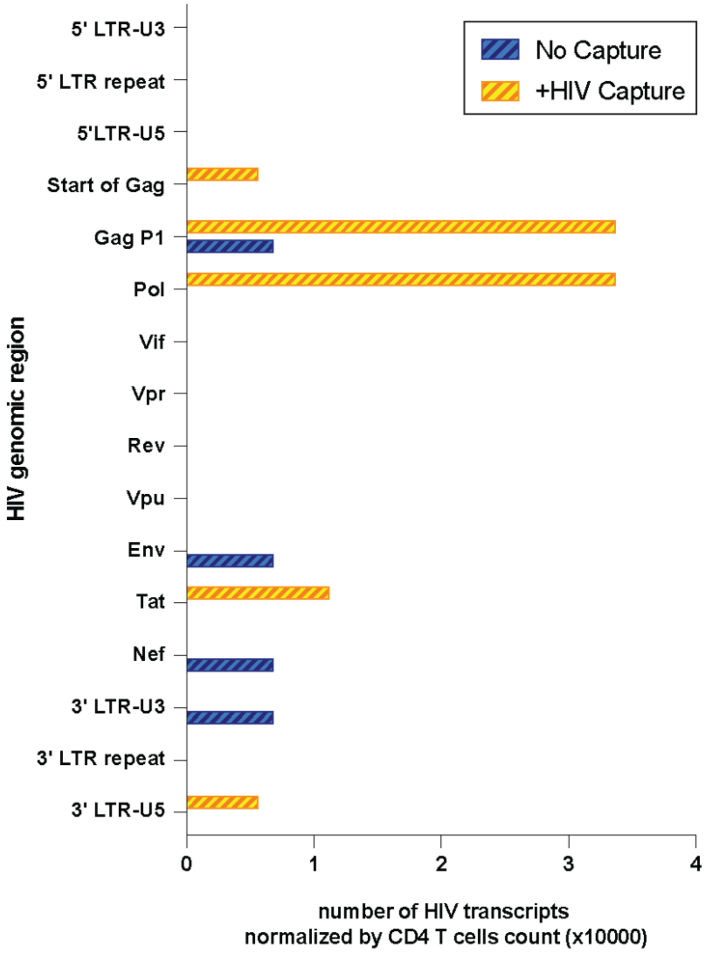
No Capture
+HIV Capture
5' LTR-U3
5' LTR repeat
5'LTR-U5
Start of Gag
Gag P1
Pol
Vif
Vpr
Rev
Vpu
Env
Tat
Nef
3' LTR-U3
3' LTR repeat
3' LTR-U5
HIV genomic region
0
1
2
3
4
number of HIV transcripts
normalized by CD4 T cells count (x10000)

97

Figure S5. HIV-seq on cells from ART-suppressed PWH increases detection of transcripts mapping to the *gag* and *pol* regions of HIV-1. HIV reads from PID1052 and PID8027 during ART suppression were aligned to the HIV-1 subtype B consensus reference genome. The y-axis depicts individual HIV genome regions, and the x-axis shows the normalized number of detected HIV transcripts per 10,000 CD4+ T cells, in the absence (blue hatched) vs. presence (yellow hatched) of the HIV capture sequences. Source data are provided as a Source Data file.

Figure S6

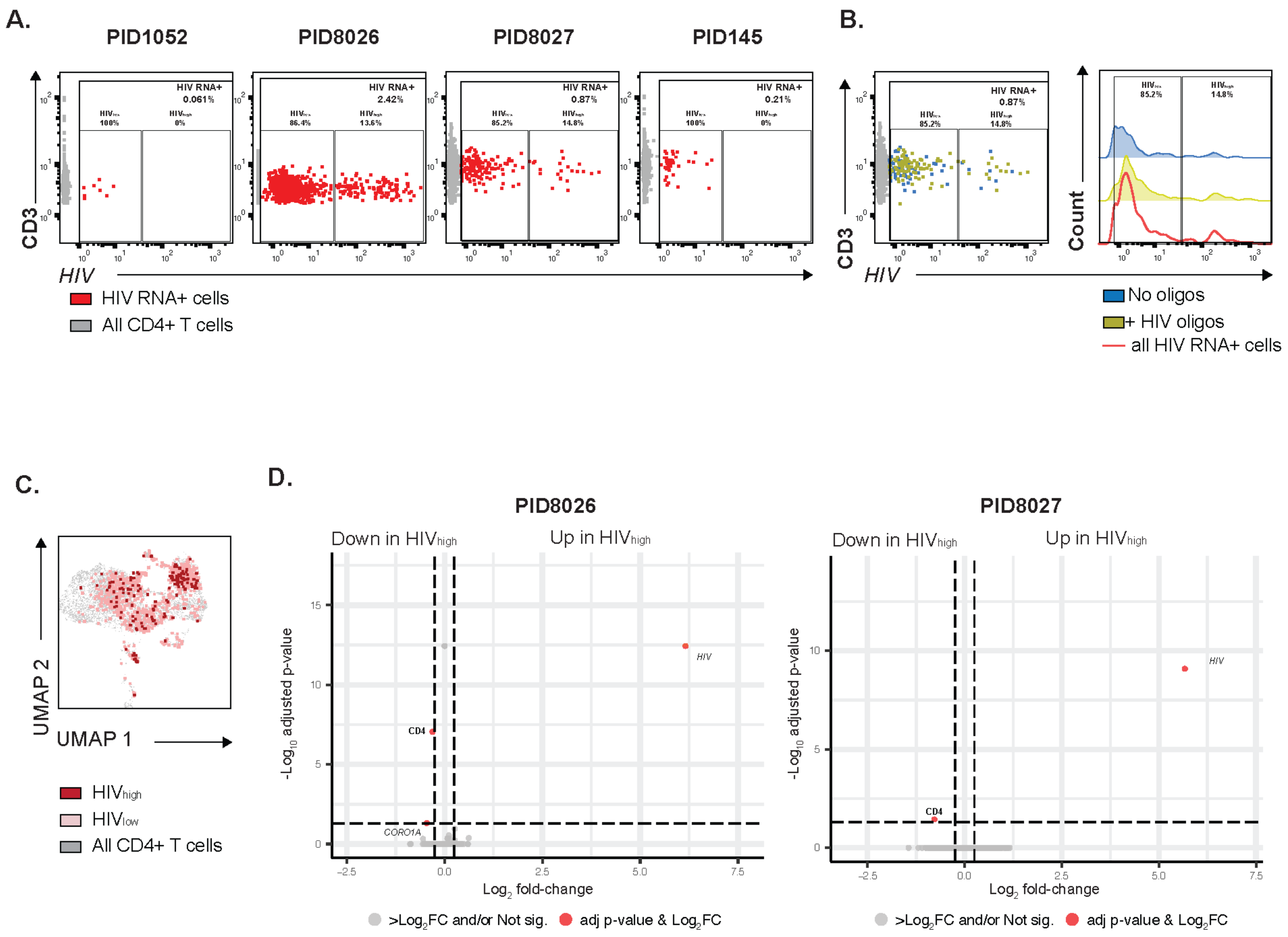
A.
PID1052
PID8026
PID8027
PID145
HIV RNA+ 0.061%
HIV RNA+ 2.42%
HIV RNA+ 0.87%
HIV RNA+ 0.21%
CD3
HIV
HIV RNA+ cells
All CD4+ T cells
B.
HIV RNA+ 0.87%
CD3
HIV
Count
No oligos
+ HIV oligos
all HIV RNA+ cells
C.
UMAP 2
UMAP 1
HIVhigh
HIVlow
All CD4+ T cells
D.
PID8026
PID8027
Down in HIVhigh
Up in HIVhigh
-Log10 adjusted p-value
Log2 fold-change
HIV
CD4
CORO1A
>Log2FC and/or Not sig.
adj p-value & Log2FC

Figure S6. HIV RNA+ cells expressing low vs. high numbers of HIV transcripts exhibit similar global gene expression profiles but differ in cell-surface CD4 expression.

A. HIV RNA+ CD4+ T cells from 4 viremic PWH can be separated into two groups: those harboring 0 to 50 HIV transcripts (HIV_{low}), and those with more than 50 HIV transcripts (HIV_{high}). HIV RNA+ cells are depicted in red and HIV RNA- cells are depicted in gray. Percentages of total HIV RNA+ cells are indicated in the upper right of each plot, and percentages of HIV_{low} and HIV_{high} populations among total HIV RNA+ cells are indicated above each population. **B.** Infected cells with high and low levels of HIV transcripts can be identified regardless of implementation of HIV-seq. HIV RNA+ cells from PID8027 were colored according to whether they were analyzed using the conventional or HIV-seq pipelines for scRNA-seq analysis. Shown on the left is a plot depicting HIV RNA+ cells in the absence (blue) vs. presence (yellow) of the HIV primers. Shown on the right is a histogram plot showing HIV RNA+ cells in the absence (blue) vs. presence (yellow) of the HIV primers, as well as the combination of HIV RNA+ cells from both conditions (red), highlighting the bimodal distribution of the HIV RNA+ cells. **C.** The transcriptomes of HIV_{low} and HIV_{high} cells are similar. Shown is a UMAP depiction of HIV_{high} cells in red and HIV_{low} cells in pink, against a background of HIV RNA- CD4+ T cells in gray. Shown are combined results for PID8026 and PID8027. **D.** HIV_{high} and HIV_{low} cells have similar transcriptomes, but HIV_{high} cells exhibit decreased cell-surface CD4 protein expression. Shown are volcano plots displaying differentially expressed transcripts and proteins for the two viremic PWH harboring both HIV_{high} and HIV_{low} cells. Select up- and down-regulated transcripts/proteins are annotated. Red dots correspond to genes and proteins with $\geq 0.25 \log_2$ fold-change expression and with p value < 0.05 (adjusted for multiple comparisons), as determined by a two-sided Wilcoxon rank sum test.

Figure S7

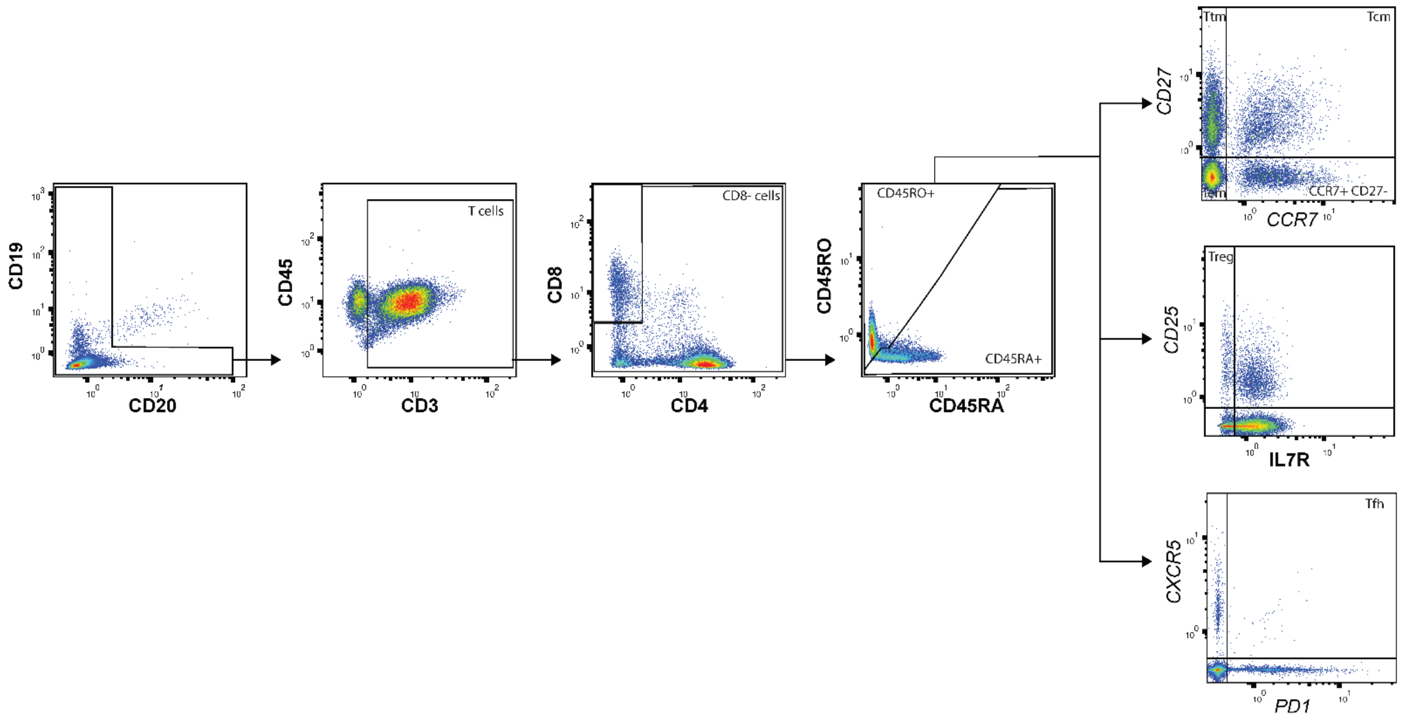

155

Figure S7. Gating strategy for CD4+ T cell subset identification. A sequential gating strategy was implemented using a combination of surface protein markers and transcripts. CD4+ T cells were defined as CD45+CD19-CD20-CD3+CD8- cells, to include HIV-infected CD4+ T cells that have downregulated cell-surface CD4. Classic CD4+ T cells subsets were then defined as follows: naïve (Tn: CD45RO-CD45RA+), central memory (Tcm: CD45RO+CD45RA-CD27+CCR7+), effector memory (Tem: CD45RO+CD45RA-CD27-CCR7-), transitional memory (Tm: CD45RO+CD45RA-CD27+CCR7-), memory CCR7+CD27-(CD45RO+CD45RA-CCR7+CD27-), regulatory T cells (Treg: CD45RO+CD45RA-CD25+IL7R-) and non-Tregs (CD45RO+CD45RA-CD25-IL7R- or CD25+IL7R+ or CD25-IL7R+), and T follicular helper (Tfh: CD45RO+CD45RA-CXCR5+PD1+) and non-Tfh (CD45RO+CD45RA-CXCR5-PD1- or CXCR5+PD1- or CXCR5-PD1+) cells. Shown are data from one representative participant (PID8027).

Figure S8

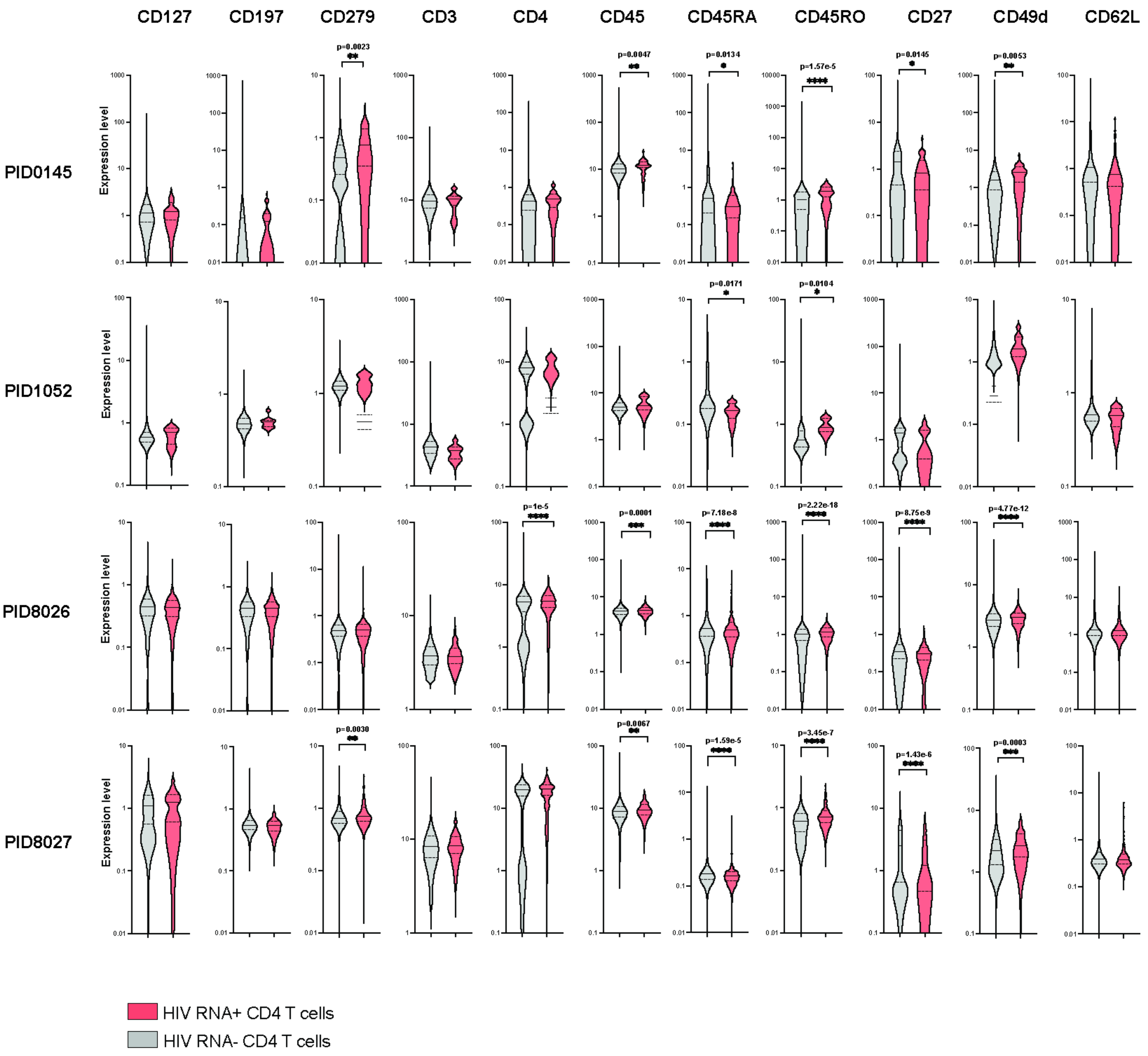

CD127
CD197
CD279
CD3
CD4
CD45
CD45RA
CD45RO
CD27
CD49d
CD62L
PID0145
PID1052
PID8026
PID8027
Expression level
p=0.0023
p=0.0047
p=0.0134
p=1.57e-5
p=0.0145
p=0.0053
p=0.0171
p=0.0104
p=1e-5
p=0.0001
p=7.18e-8
p=2.22e-18
p=8.75e-9
p=4.77e-12
p=0.0030
p=0.0067
p=1.59e-5
p=3.45e-7
p=1.43e-6
p=0.0003
HIV RNA+ CD4 T cells
HIV RNA- CD4 T cells

Figure S8. Compared to HIV RNA- cells, HIV RNA+ cells during viremia show higher levels of protein markers associated with memory CD4+ T cells.

Violin plots depict relative expression levels (log10 scale) of 11 surface markers (CD127, CD197, CD279, CD3, CD4, CD45, CD45RA, CD45RO, CD27, CD49D, CD62L) on CD4+ T cells from four individual PWH (PID0145, PID1052, PID8026 and PID8027) during viremia. Expression levels were determined from CITE-seq data. Plots depict HIV RNA- CD4+ T cells (gray) and HIV RNA+ CD4+ T cells (red) from the same person. N=19,020, 13,027, 34,980 and 17,568 HIV RNA- CD4+ T cells and 40, 8, 869 and 155 HIV RNA+ cells for PID145, PID1052, PID8026 and PID8027, respectively. Thick black bars indicate the median, and thin black bars represent the interquartile range. *P<0.05, **P<0.01, ***P<0.001, ****P 0.0001, as determined by a two-sided Mann–Whitney test with FDR correction for multiple comparisons. Exact P values are indicated on the graph. Source data are provided as a Source Data file.

Figure S9

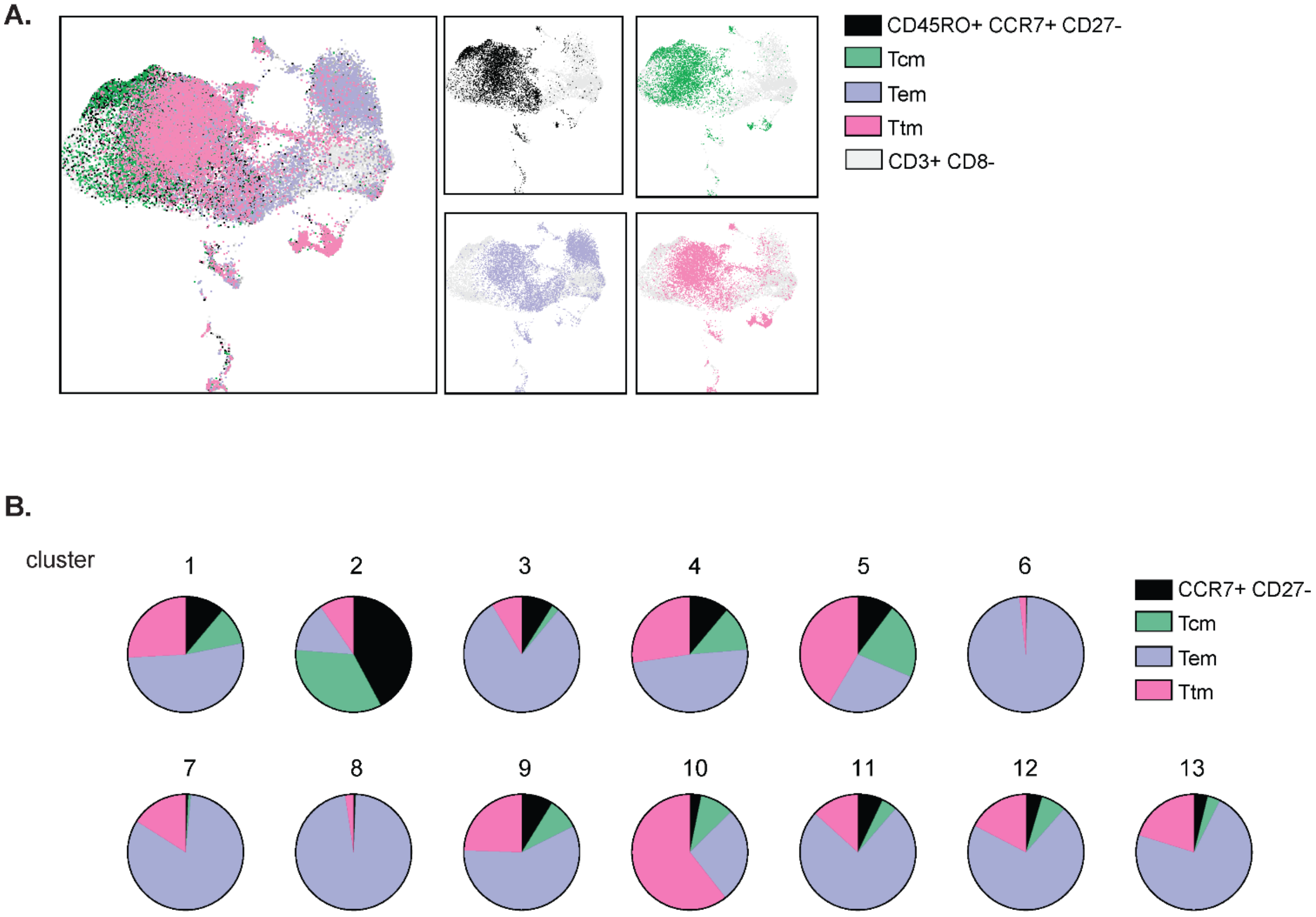

213

Figure S9. Distribution of classic memory CD4+ T cell subsets among clusters from viremic PWH. A. UMAP depicting classic memory CD4+ T subsets (Tcm, Tem, Ttm, and CCR7+CD27- memory cells) among CD3+ CD8- cells. **B.** Shown are pie graphs depicting the proportion of classic memory CD4+ T subsets (Tcm, Tem, Ttm, and CCR7+CD27- memory cells) among the 13 clusters identified by scRNA-seq in specimens from viremic PWH. Source data are provided as a Source Data file.

Figure S10

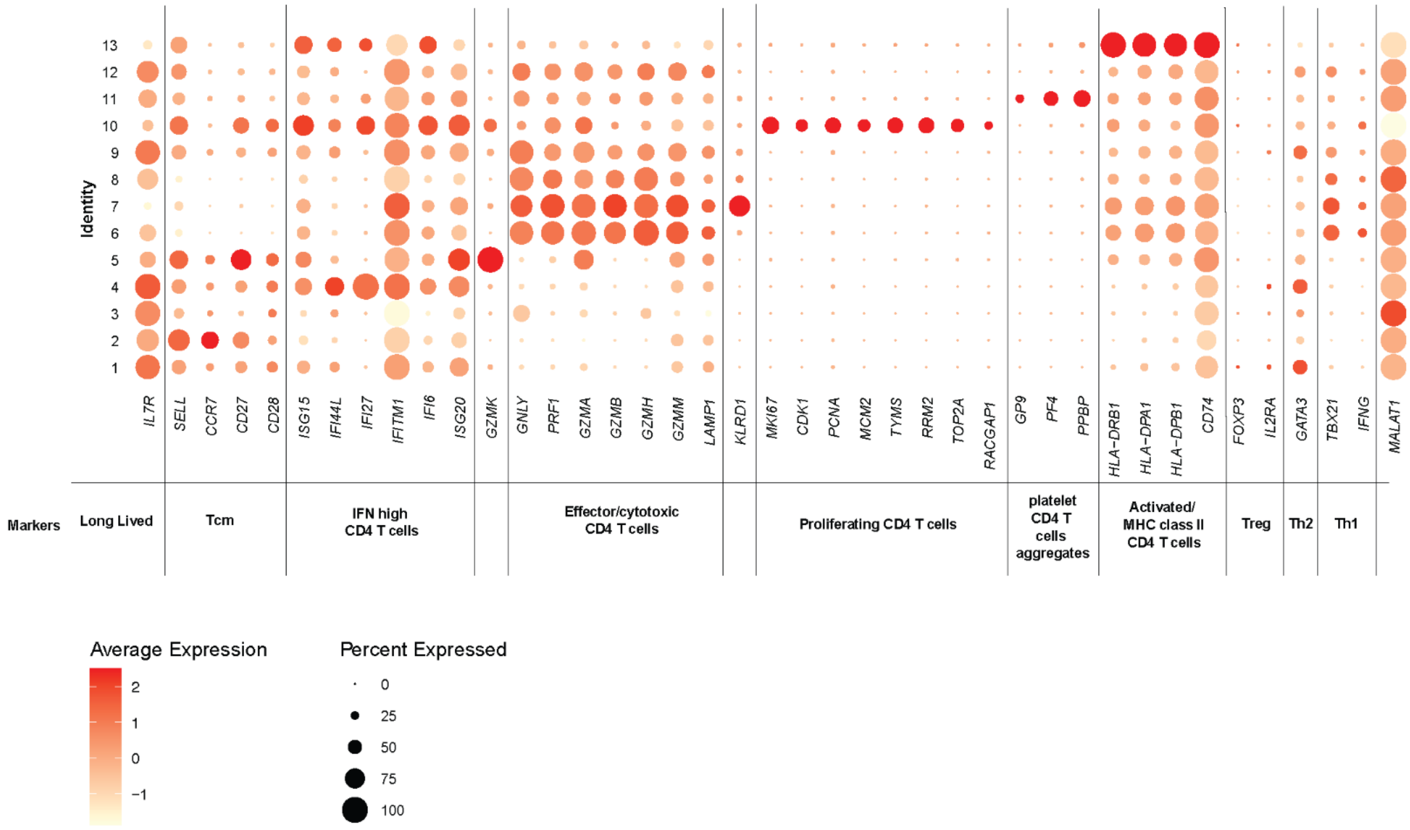
Identity
13
12
11
10
9
8
7
6
5
4
3
2
1
IL7R
SELL
CCR7
CD27
CD28
ISG15
IFI44L
IFI27
IFITM1
IFI6
ISG20
GZMK
GNLY
PRF1
GZMA
GZMB
GZMH
GZMM
LAMP1
KLRD1
MKI67
CDK1
PCNA
MCM2
TYMS
RRM2
TOP2A
RACGAP1
GP9
PF4
PPBP
HLA−DRB1
HLA−DPA1
HLA−DPB1
CD74
FOXP3
IL2RA
GATA3
TBX21
IFNG
MALAT1
Markers
Long Lived
Tcm
IFN high CD4 T cells
Effector/cytotoxic CD4 T cells
Proliferating CD4 T cells
platelet CD4 T cells aggregates
Activated/ MHC class II CD4 T cells
Treg
Th2
Th1
Average Expression
2
1
0
−1
Percent Expressed
0
25
50
75
100

Figure S10. Cluster annotation.

Dot plot displaying level of expression of genes used to annotate the thirteen clusters of CD4+ T cells. Cluster 1 expressed the long-lived marker *IL7R* and high levels of the Th2 lineage factor *GATA3*. Cluster 2 expressed markers of Tcm cells (high *SELL, CCR7,* and *CD27*). Cluster 3 expressed *IL7R* and was defined by the highest expression of the lncRNA *MALAT1*. Cluster 4 expressed *IL7R* and a large collection of interferon-stimulated genes (high *ISG15, IFI44L, IFI27, IFITM1, IFI6,* and *ISG20*). Cluster 5 corresponded to Ttm (low *CCR7*, high *CD27*) and was distinguished by high expression of *GZMK*. Cluster 6 cells were Tem (low *SELL, CCR7,* and *CD27*) with a cytotoxic signature (high *GNLY, PRF1, GZMA, GZMB, GZMH, GZMM,* and *LAMP1*) and expressed Th1-associated genes (high *TBX21* and *IFNG*). Cluster 7 also exhibited a cytotoxic signature and was distinguished by high expression of the inhibitory receptor *KLRD1*. Cluster 8 comprised a second cluster of Tem cells expressing cytotoxic markers. Cluster 9 expressed high levels of *IL7R* alongside cytotoxic markers. Cluster 10 expressed both interferon-stimulated genes and proliferation markers (high *MKI67, CDK1, PCNA, MCM2, TYMS, RRM2, TOP2A, RACGAP1*). Cluster 11 expressed markers of a previously described[111] platelet–CD4+ T cell aggregate population (high *GP9, PF4, PPBP*. Cluster 12 did not display a readily definable transcriptional signature and was annotated as other CD4+ T cells. Cluster 13 cells were activated cells that expressed genes involved in MHC class II antigen presentation (high *HLA-DRB1, HLA-DPA1, HLA-DPB1*, and *CD74*). Dot sizes indicate percentage of cells in a cluster expressing each gene; dot colors reflect expression level. Cluster numbers are indicated on the left.

Figure S11

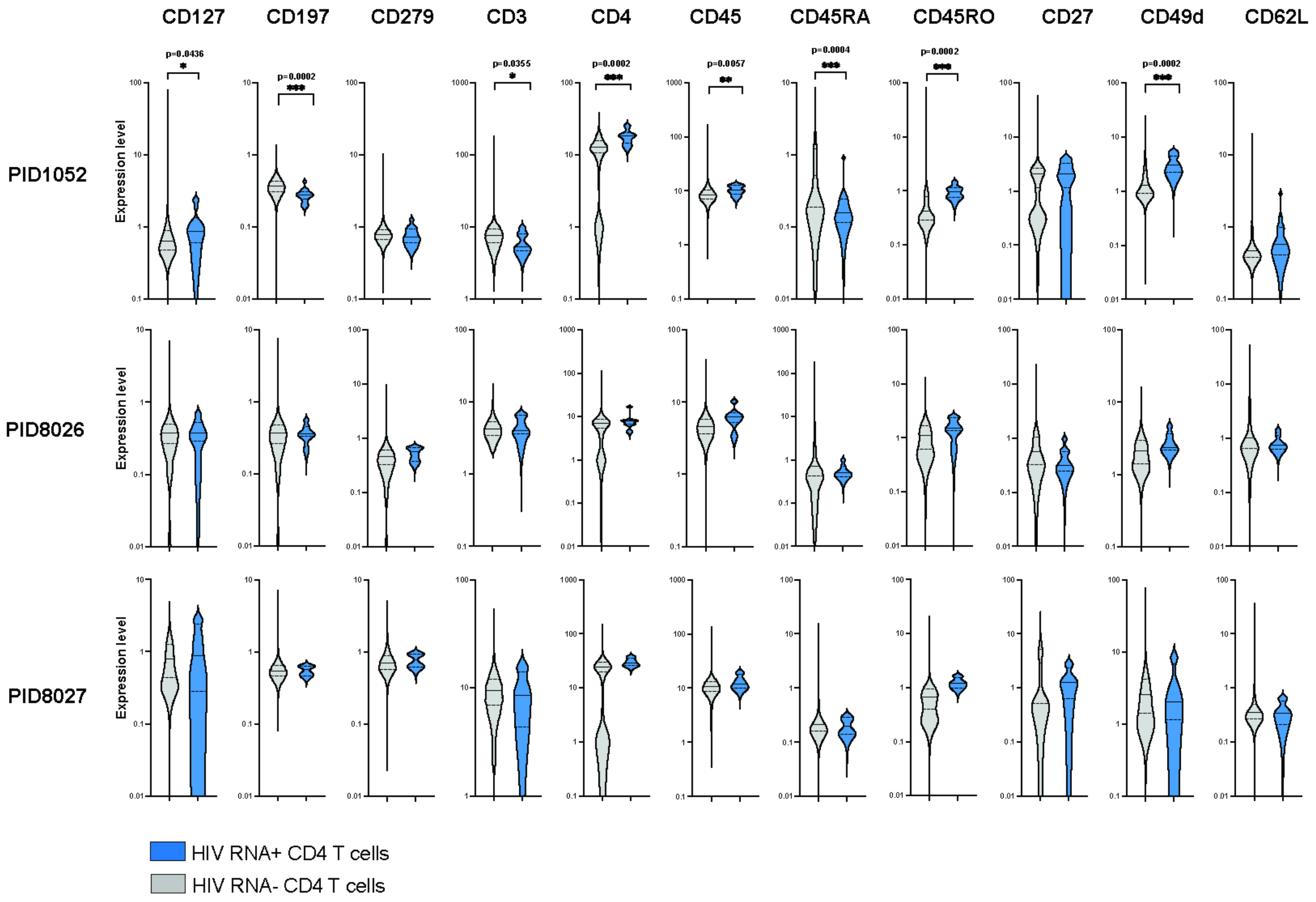
CD127
CD197
CD279
CD3
CD4
CD45
CD45RA
CD45RO
CD27
CD49d
CD62L
PID1052
PID8026
PID8027
Expression level
p=0.0436
p=0.0002
p=0.0355
p=0.0002
p=0.0057
p=0.0004
p=0.0002
p=0.0002
HIV RNA+ CD4 T cells
HIV RNA- CD4 T cells

Figure S11. Differential Protein Expression analysis between HIV RNA- cells and HIV RNA+ cells during ART suppression.

Violin plots depict relative expression levels (log10 scale) of 11 surface markers (CD127, CD197, CD279, CD3, CD4, CD45, CD45RA, CD45RO, CD27, CD49D, CD62L) on CD4+ T cells from three individual PWH (PID1052, PID8026 and PID8027). Expression levels were determined from CITE-seq data. Plots depict HIV RNA- CD4+ T cells (gray) and HIV RNA+ CD4+ T cells (blue) from the same donor. N= 15,454, 42,611 and 17,068 HIV RNA- CD4+ T cells and 14, 7 and 4 HIV RNA+ cells for PID1052, PID8026 and PID8027, respectively. Thick black bars indicate the median, and thin black bars represent the interquartile range. *P<0.05, **P<0.01, ***P<0.001, ****P<0.0001, as determined by a two-sided Mann–Whitney test with FDR correction for multiple comparisons. Exact P values are indicated on the graph. Source data are provided as a Source Data file.

Figure S12

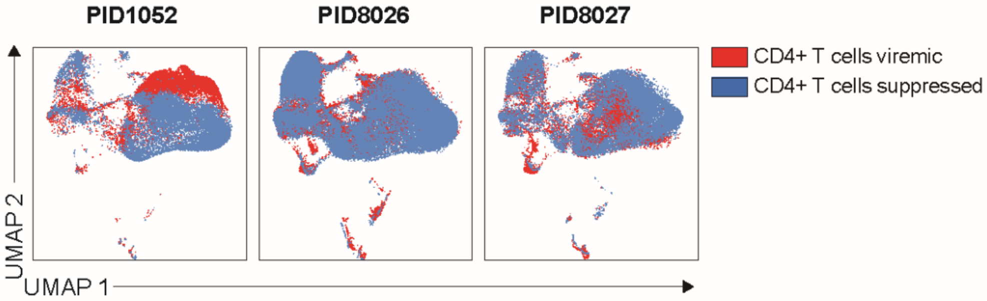
PID1052
PID8026
PID8027
CD4+ T cells viremic
CD4+ T cells suppressed
UMAP 2
UMAP 1

Figure S12. ART suppression elicits global changes in host transcriptome. Shown are UMAP plots depicting total CD4+ T cells from viremic (red) and suppressed (blue) time points for each of the three PWH with paired specimens (PID1052, PID8026, and PID8027), after applying Harmony batch correction. For all three participants, CD4+ T cells during viremia localize in different regions of the UMAP relative to CD4+ T cells during ART suppression.

Figure S13

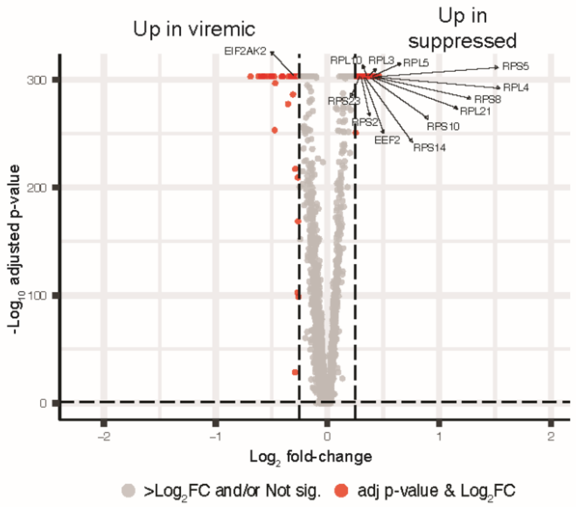

Up in viremic
Up in suppressed
EIF2AK2
RPL10
RPL3
RPL5
RPS5
RPL4
RPS8
RPL21
RPS10
RPS14
EEF2
RPS2
RPS23
-Log10 adjusted p-value
300
200
100
0
-2
-1
0
1
2
Log2 fold-change
>Log2FC and/or Not sig.
adj p-value & Log2FC

Figure S13. Compared to CD4+ T cells during ART, CD4+ T cells during viremia exhibit an activation of the integrated stress pathway with diminished ribosomal transcript expression levels. Shown is a volcano plot displaying differentially expressed transcripts in CD4+ T cells from viremic vs. suppressed time points, with ribosomal transcripts and transcripts related to ISR pathway annotated. Red dots correspond to transcripts with ≥0.25$\log_2$ fold-change expression and p value <0.05 (adjusted for multiple comparisons), as determined by the two-sided Wilcoxon rank sum test.

Figure S14

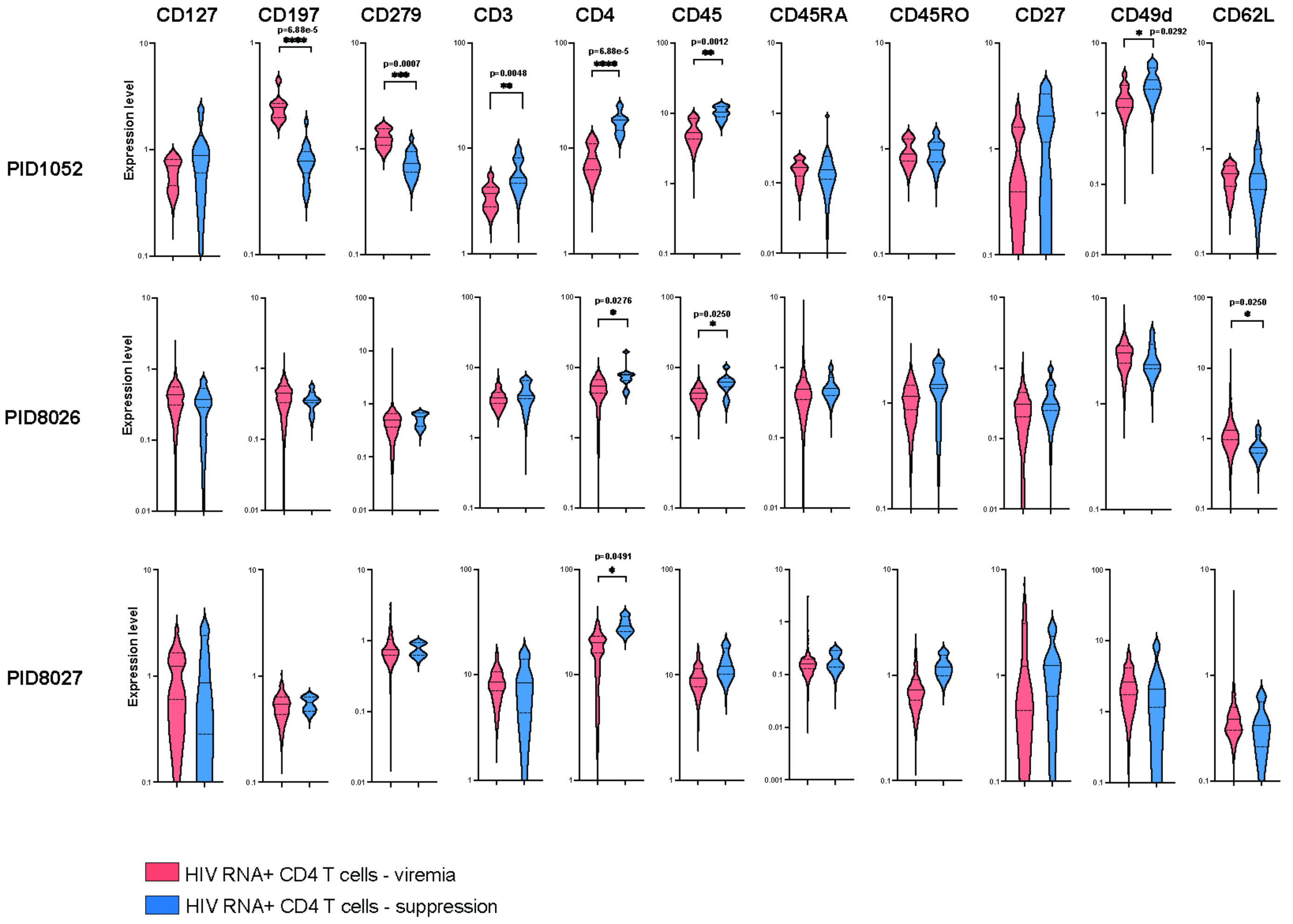
CD127
CD197
CD279
CD3
CD4
CD45
CD45RA
CD45RO
CD27
CD49d
CD62L
PID1052
PID8026
PID8027
Expression level
p=6.88e-5
p=0.0007
p=0.0048
p=6.88e-5
p=0.0012
p=0.0292
p=0.0276
p=0.0250
p=0.0250
p=0.0491
HIV RNA+ CD4 T cells - viremia
HIV RNA+ CD4 T cells - suppression

Figure S14. During viremia, HIV RNA+ cells express lower levels of cell-surface CD4 protein compared to HIV RNA+ cells during ART suppression.

Violin plots depict relative expression levels (log10 scale) of 11 different surface markers (CD127, CD197, CD279, CD3, CD4, CD45, CD45RA, CD45RO, CD27, CD49D, CD62L) on HIV RNA+ CD4 T cells from three PWH (PID1052, PID8026 and PID8027). Plots depict HIV RNA+ CD4+ T cells during viremia (red) and HIV RNA+ CD4+ T cells during ART suppression (blue) from the same person. N= 6, 869 and 155 HIV RNA+ CD4+ T cells during viremia and 14, 7 and 4 HIV RNA+ cells during ART suppression for PID1052, PID8026 and PID8027, respectively. Thick black bars indicate the median, and thin black bars represent the interquartile range. *P<0.05, **P<0.01, ***P 0.001, ****P<0.0001, as determined by a two-sided Mann–Whitney test with FDR correction for multiple comparisons. Exact P values are indicated on the graph. Source data are provided as a Source Data file.
