# Supplementary material for: HIV-seq reveals gene expression differences between HIV-transcribing cells from viremic and suppressed people with HIV
Source: Nat Commun. 2026 Mar 3;17:1540. doi: 10.1038/s41467-026-68797-3 (PMC12957429; doi:10.1038/s41467-026-68797-3)
Supplement: Supplementary file 2 — Description of Additional Supplementary Files [file 41467_2026_68797_MOESM2_ESM.pdf]

Description of Additional Supplementary Files

File Name: Supplementary Data 1
Description: TotalSeq C antibody panel for CITE-seq analysis

File Name: Supplementary Data 2
Description: HIV-seq capture sequences

File Name: Supplementary Data 3
Description: Differentially expressed genes for PID1052 during viremia of CD4+ T cells processed with and without HIV capture sequences identified using two-sided Wilcoxon Rank-sum test (adjusted p-value < 0.05 and log2(fold-change) >0.25)

File Name: Supplementary Data 4
Description: Differentially expressed genes for PID8027 during viremia of CD4+ T cells processed with and without HIV capture sequences identified using two-sided Wilcoxon Rank-sum test (adjusted p-value < 0.05 and log2(fold-change) >0.25)

File Name: Supplementary Data 5
Description: Differentially expressed genes between HIV RNA+ cells vs. HIV RNA- cells during viremia (n=4) determined by two-sided gene-wise quasi F-tests on the samples' pseudo-bulked gene counts (adjusted p-value < 0.05 and log2(fold-change) > 0.25)

File Name: Supplementary Data 6
Description: Percentages of cells belonging to each cluster during virenia and suppression

File Name: Supplementary Data 7
Description: Differentially expressed genes between HIV RNA+ cells vs. HIV RNA- cells during ART suppression (n=3) determined by two-sided genewise quasi F-tests on the samples' pseudo-bulked gene counts (adjusted p-value < 0.05 and log2(fold-change) > 0.25)

File Name: Supplementary Data 8
Description: Differentially expressed genes in paired samples of CD4+ T cells from during viremia vs. ART suppression (n=3) determined by two-sided genewise quasi F-tests on the samples' pseudo-bulked gene counts (p-value < 0.05 and log2(fold-change) > 0.25)

File Name: Supplementary Data 9
Description: Differentially expressed genes in paired samples of CD4+ T cells from during viremia vs. ART suppression (n=3) identified using two-sided Wilcoxon Rank-sum test (adjusted p-value < 0.05 and log2(fold-change) >0.25)

File Name: Supplementary Data 10
Description: Differentially expressed genes in paired samples of HIV RNA+ CD4+ T cells from during viremia vs. ART suppression (n=3) determined by two-sided genewise quasi F-tests on the samples' pseudo-bulked gene counts (p-value < 0.05 and log2(fold-change) >0.25)
