# Supplementary material for: HIV-seq reveals gene expression differences between HIV-transcribing cells from viremic and suppressed people with HIV
Source: Nat Commun. 2026 Mar 3;17:1540. doi: 10.1038/s41467-026-68797-3 (PMC12957429; doi:10.1038/s41467-026-68797-3)
Supplement: Supplementary file 4 — Reporting Summary [file 41467_2026_68797_MOESM4_ESM.pdf]

nature portfolio

Corresponding author(s): Steven Yukl, Nadia Roan

Last updated by author(s): Dec 17, 2025

Reporting Summary

Nature Portfolio wishes to improve the reproducibility of the work that we publish. This form provides structure for consistency and transparency in reporting. For further information on Nature Portfolio policies, see our Editorial Policies and the Editorial Policy Checklist.

Statistics

For all statistical analyses, confirm that the following items are present in the figure legend, table legend, main text, or Methods section.

n/a	Confirmed	
☐	☒	The exact sample size (*n*) for each experimental group/condition, given as a discrete number and unit of measurement
☐	☒	A statement on whether measurements were taken from distinct samples or whether the same sample was measured repeatedly
☐	☒	The statistical test(s) used AND whether they are one- or two-sided<br>*Only common tests should be described solely by name; describe more complex techniques in the Methods section.*
☒	☐	A description of all covariates tested
☐	☒	A description of any assumptions or corrections, such as tests of normality and adjustment for multiple comparisons
☐	☒	A full description of the statistical parameters including central tendency (e.g. means) or other basic estimates (e.g. regression coefficient) AND variation (e.g. standard deviation) or associated estimates of uncertainty (e.g. confidence intervals)
☐	☒	For null hypothesis testing, the test statistic (e.g. *F*, *t*, *r*) with confidence intervals, effect sizes, degrees of freedom and *P* value noted<br>*Give P values as exact values whenever suitable.*
☒	☐	For Bayesian analysis, information on the choice of priors and Markov chain Monte Carlo settings
☒	☐	For hierarchical and complex designs, identification of the appropriate level for tests and full reporting of outcomes
☒	☐	Estimates of effect sizes (e.g. Cohen's *d*, Pearson's *r*), indicating how they were calculated

Our web collection on statistics for biologists contains articles on many of the points above.

Software and code

Policy information about availability of computer code

Data collection	No software was used
Data analysis	Commercial software was used, including: the Los Alamos National Laboratory's HIV sequence database (2018 version); MAFFT; Geneious; Cell Ranger v6.0.2; DoubletFinder (in R); Seurat NormalizeData (from Seurat package, in R); SeqGeq; Seurat v4.3.0; FindClusters Seurat; Ime4 (in R); emmeans (in R); muscat (in R); Enrichr. All code used to generate results has been deposited on Github (https://github.com/gladstone-institutes/HIV-seq-_Viremic_vs._ART) and has been archived on Zenodo for reproducibility[111](Gill N., 2025; https://doi.org/10.5281/zenodo.17886108).

For manuscripts utilizing custom algorithms or software that are central to the research but not yet described in published literature, software must be made available to editors and reviewers. We strongly encourage code deposition in a community repository (e.g. GitHub). See the Nature Portfolio guidelines for submitting code & software for further information.

Data

Policy information about availability of data

All manuscripts must include a data availability statement. This statement should provide the following information, where applicable:

- Accession codes, unique identifiers, or web links for publicly available datasets
- A description of any restrictions on data availability
- For clinical datasets or third party data, please ensure that the statement adheres to our policy

The sequence datasets generated during this study are available at GEO repository GSE266455 (for PID 0145, 8026, 8027 and 1052; https://www.ncbi.nlm.nih.gov/

geo/query/acc.cgi?acc=GSE266455; enter token azodqesezbelnuj into the box) and at GSE305352 (for datasets generated from people without HIV; https://www.ncbi.nlm.nih.gov/geo/query/acc.cgi?acc=GSE305352; enter token ulwfmyesxxgtjgn into the box).

Research involving human participants, their data, or biological material

Policy information about studies with human participants or human data. See also policy information about sex, gender (identity/presentation), and sexual orientation and race, ethnicity and racism.

Reporting on sex and gender	Study participant numbers, gender, and age are listed in Table 1, along with other relevant demographic and clinical information. In this pilot study where it was only feasible to study a small number of participants, and given the difficulty in finding paired longitudinal samples from before and after ART, we asked for any available samples, which were provided to us in de-identified form. Therefore, sex/gender was not considered in the study design. Data on sex/gender was obtained after completion of data analysis, and was self-reported. Of the four participants with HIV, three were male and one was female. With only three male participants and one female participant, the numbers were deemed insufficient for a post-hoc analysis.
Reporting on race, ethnicity, or other socially relevant groupings	The study participants were people with HIV on suppressive ART from diverse demographic and clinical backgrounds.<br>The gender, race/ethnicity, and age range of each participant are indicated in the manuscript, but those variables were not considered as biological variables in this study.
Population characteristics	Two participants were recruited from the SCOPE cohort. SCOPE is an observational, prospective study of HIV-1 infected volunteers designed to provide a specimen bank of samples with carefully characterized clinical data. The cohort is composed of well characterized people with HIV and uninfected persons of diverse demographic and clinical backgrounds.<br><br>One participant was collected from the Treat Acute cohort, which is a cohort of people with HIV who have been recruited and treated during acute HIV infection, within days or few weeks after exposure to the virus. The cohort is composed of well characterized persons of diverse demographic and clinical backgrounds.<br><br>One participant was recruited before ART initiation from the San Francisco VA Medical Center.
Recruitment	Participants were seen at San Francisco General Hospital or at the San Francisco VA Medical Center. Each visit includes a confidential and detailed interview as well as a blood draw.
Ethics oversight	The study was approved by the Committee on Human Research (CHR), the Institutional Review Board for the University of California, San Francisco (approval #11–07551 and #10-01561). All study participants provided written informed consent.

Note that full information on the approval of the study protocol must also be provided in the manuscript.

Field-specific reporting

Please select the one below that is the best fit for your research. If you are not sure, read the appropriate sections before making your selection.

[x] Life sciences   [ ] Behavioural & social sciences   [ ] Ecological, evolutionary & environmental sciences

For a reference copy of the document with all sections, see nature.com/documents/nr-reporting-summary-flat.pdf

Life sciences study design

All studies must disclose on these points even when the disclosure is negative.

Sample size	The sample size was determined based on the limited availability of rare clinical samples from people living with HIV (matched longitudinal blood samples from viremic and ART-suppressed timepoints) and the exploratory nature of the study. While the number of samples was small, the depth of single-cell RNA sequencing, with at least 50,000 reads per cell for the GEX library, and 13,000 to 42,000 cells sequenced per sample, allowed for robust characterization of cellular heterogeneity and significant biological insights.
Data exclusions	No data were excluded from the analysis.
Replication	To verify the reproducibility of our experimental findings, we utilized biological replicates from up to four different people living with HIV (PWH) and three different HIV- blood donors to enhance the reliability and generalizability of our results. We employed statistical analyses (as detailed in the Method section of the manuscript) to take into account the variability across these samples, ensuring that our conclusions are robust. The key findings were reproducible across study participants. Given the extremely high cost and time required for these single cell studies, it was not feasible for us to study more participants.
Randomization	Participants were selected based on availability of cryopreserved PBMC from before the start of ART and a preference for a longitudinal sample of cryopreserved PBMC obtained after ART suppression. PBMC samples from before ART are not usually available, and it is even more difficult to find paired longitudinal samples from before and after ART. The study designers saw no clear role for randomization.
Blinding	Blinding was not employed in this study, as the scRNA-seq was conducted on known rare specimens. Given the nature of the analysis, where the focus is on unbiased profiling of gene expression at the single-cell level, the absence of blinding is not expected to impact the reliability of the results.

Reporting for specific materials, systems and methods

We require information from authors about some types of materials, experimental systems and methods used in many studies. Here, indicate whether each material, system or method listed is relevant to your study. If you are not sure if a list item applies to your research, read the appropriate section before selecting a response.

Materials & experimental systems

n/a	Involved in the study	
☐	☒	Antibodies
☒	☐	Eukaryotic cell lines
☒	☐	Palaeontology and archaeology
☒	☐	Animals and other organisms
☐	☒	Clinical data
☒	☐	Dual use research of concern
☒	☐	Plants

Methods

n/a	Involved in the study	
☒	☐	ChIP-seq
☒	☐	Flow cytometry
☒	☐	MRI-based neuroimaging

Antibodies

Antibodies used	TotalSeq-C pooled antibody mix (from Biolegend) was used, and detailed information about the mix is provided in the Methods and Supplementary Table 1. The supplier name, catalog number, clone name, and final staining concentration are provided in Supplementary Table 1.
Validation	Each antibody has been validated by the manufacturer as stated in their technical data sheet: "Each lot of this antibody is quality control tested by immunofluorescent staining with flow cytometric analysis and the oligomer sequence is confirmed by sequencing." All antibodies were used at a final concentration that was established during internal optimization of the staining panel on unpublished samples. Validation was performed at the panel level rather than for each antibody individually, and the selected concentration consistently yielded specific and reproducible detection of all targets.

Clinical data

Policy information about clinical studies

All manuscripts should comply with the ICMJE guidelines for publication of clinical research and a completed CONSORT checklist must be included with all submissions.

Clinical trial registration	N/A
Study protocol	N/A
Data collection	N/A
Outcomes	N/A

Plants

Seed stocks	N/A
Novel plant genotypes	N/A
Authentication	N/A
